# A Revised Stem Cell Theory for the Pathogenesis of Endometriosis

**DOI:** 10.3390/jpm12020216

**Published:** 2022-02-04

**Authors:** Tetsuo Maruyama

**Affiliations:** Department of Obstetrics and Gynecology, Keio University School of Medicine, Tokyo 160-8582, Japan; te-suo@keio.jp

**Keywords:** endometriosis, stem cells, endometrium, pathogenesis, origin

## Abstract

During the past decade, a stem cell-based hypothesis has emerged (among many others) to explain the pathogenesis of endometriosis. The initial hypothesis proposed that endometriosis arose from a single or a few specific cells with stem cell properties, including self-renewal and multi-lineage cell differentiation. The origins of the endometriosis-initiating stem cells were thought to be the bone marrow, uterine endometrium, and other tissues. Based on the implantation or metastatic theory in combination with the initial stem cell theory, one or a few multipotent stem/progenitor cells present in the eutopic endometrium or bone marrow translocate to ectopic sites via fallopian tubes during menstruation, vasculolymphatic routes, or through direct migration and invasion. Subsequently, they give rise to endometriotic lesions followed by differentiation into various cell components of endometriosis, including glandular and stromal cells. Recent somatic mutation analyses of deep infiltrating endometriosis, endometrioma, and eutopic normal endometrium using next-generation sequencing techniques have redefined the stem cell theory. It is now proposed that stem/progenitor cells of at least two different origins—epithelium and stroma—sequentially, differentially, but coordinately contribute to the genesis of endometriosis. The dual stem cell theory on how two (or more) stem/progenitor cells differentially and coordinately participate in the establishment of endometriotic lesions remains to be elucidated. Furthermore, the stem/progenitor cells involved in this theory also remain to be identified. Given that the origin of endometriosis is eutopic endometrium, the candidate cells for endometriotic epithelium-initiating cells are likely to be endometrial epithelial cells positive for either N-cadherin or SSEA-1 or both. The candidate cells for endometriotic stroma-initiating cells may be endometrial mesenchymal stem cells positive for SUSD2. Endometrial side population cells are also a possible candidate because they contain unipotent or multipotent cells capable of behaving as endometrial epithelial and stromal stem/progenitor cells.

## 1. Introduction

Many hypotheses explaining the pathogenesis of endometriosis have been proposed over the past 100 years. The retrograde menstruation theory accounts for the pathogenesis of various types of endometrioses, and it is the most widely accepted among the various hypotheses. They include the coelomic metaplastic theory, embryonic rest theory, and lymphovascular metastasis theory [1,2,3,4,5]. According to the retrograde menstruation theory, implantation of eutopic endometrial tissue rather than cells results in the generation of endometriotic lesions. However, endometriosis is found, though very rarely, in premenarcheal girls who have experienced no menstruation [6,7] and even in the female fetus [8] and men [9,10]. Furthermore, despite the high prevalence of peritoneal endometriosis, it is very rare to microscopically detect the initial steps of endometrial tissue implants, including the attachment of the endometrial tissue to the peritoneum and its secondary proliferation and invasion [3,11,12].

Thus, the retrograde menstruation theory alone cannot perfectly account for the pathogenesis of all types of endometrioses, although endometriosis in premarcheal girls is thought to result from the seeding of naive endometrial progenitor cells into the pelvic cavity at the time of neonatal uterine bleeding [13]. The weakness of the retrograde menstruation theory, in turn, further substantiates the coelomic metaplastic theory or embryonic rest theory in which endometriosis metaplastically arises from specialized extra-uterine cells present in the mesothelial lining of the visceral and abdominal peritoneum or residual embryonic cells of the Wolffian or Mullerian ducts [1,2,3,4,5]. Each hypothesis alone is insufficient to explain the pathogenesis of all types of endometrioses. Therefore, a combination of multiple theories may be needed to explain the pathologies observed. Thus, a single hypothesis appropriate for a specific type of endometriosis—retrograde menstruation theory for peritoneal endometriosis, coelomic metaplastic theory for ovarian endometrioma, and embryo rest theory for deep infiltrating endometriosis [14]—has been explored to account for the pathogenesis of endometriosis, but these attempts have not been fully successful.

Most of the current theories have been based on the assumption that endometriotic cells originate from the eutopic endometrium. However, other theories are suggested by new advances in endometrial physiology. For example, over the past two decades, endometrial stem cell physiology has been extensively explored through identification, isolation, and characterization of endometrial stem/progenitor cells and clarification of their roles in endometrial structure and function [15,16,17,18,19,20]. These advancements led to the initial stem cell theory for the pathogenesis of endometriosis [4,15,16,21], providing new insights that explain the pathogenesis of almost all types of endometrioses in combination with retrograde menstruation- and lymphovascular metastasis-driven implantation theories [4].

## 2. Endometrial Stem/Progenitor Cells

Since the first discovery of ways to derive embryonic stem cells from early mouse embryos [22], stem cell biology and regenerative medicine have dramatically advanced our understanding of stem/progenitor cells and their roles in a variety of tissues and organs, including female reproductive organs [15,16,23,24]. Tissue somatic cells (adult stem cells) are able to self-renew indefinitely and multiply by asymmetric cell division, eventually giving rise to all cell types of the organ/tissue from which they originate through terminal differentiation [25]. Tissue stem cells play critical role(s) in the maintenance of organ structure and function in that they are required for the replacement of dying cells within organs and for tissue regeneration following physiological and pathological injuries [26,27].

**Figure 1:**
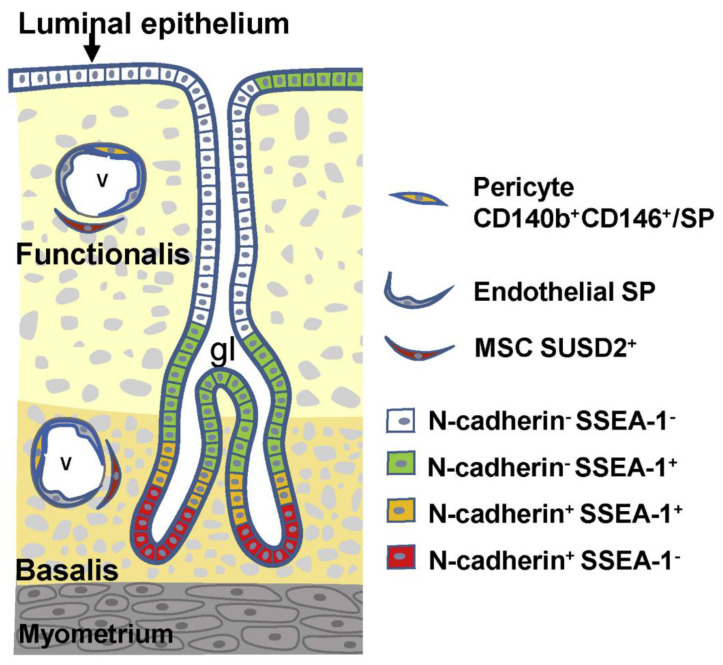
Localization and markers of human endometrial stem/progenitor cells. Endometrial stromal (mesenchymal) stem/progenitor cells are divided into three types: (1) pericytes doubly positive for CD140b and CD146 or those with SP phenotype, (2) endothelial SP, and (3) perivascular SUSD2^+^ (sushi domain-containing 2) cells. Endometrial epithelial stem/progenitor cells are divided into at least four types based on the expression pattern of N-cadherin and SSEA-1 (stage specific embryonic antigen-1). There may exist an epithelial cell hierarchy in which N-cadherin^+^SSEA-1^−^ cells (red) are the most primitive, followed by a hierarchy as follows: N-cadherin^+^SSEA-1^+^, N-cadherin^−^SSEA-1^+^, and N-cadherin^−^SSEA-1^−^. The surface markers of endometrial epithelial stem/progenitor cells have been further characterized in detail [20]. Reproduced from [19] with permission from Elsevier Press.

The human endometrium undergoes cyclic repetitive changes that include proliferation, differentiation, tissue breakdown, and shedding (menstruation) more than 400 times throughout a woman’s reproductive life [28]. The repetitive changes are ovarian steroid hormone-dependent [28] and can be reproduced in vivo using mouse models of endometrial tissue regeneration [29]. Based on those features, one or more endometrium-specific tissue stem cell systems are believed to be critical for the regeneration and remodeling properties of the endometrium [30,31]. For the past 20 years, several laboratories, including ours, have used a variety of methods to identify, isolate, and/or characterize putative endometrial stem/progenitor cells, including endometrial mesenchymal stem cells, endometrial epithelial stem/progenitor cells, and endometrial side population (ESP) cells [15,16,17,18,19,20]. Figure 1 illustrates the current understanding of the localization and markers of these human endometrial stem/progenitor cells [19]. Recently, the surface markers of endometrial epithelial stem/progenitor cells have been characterized in greater detail [20]. Importantly, in addition to the cells present in the eutopic endometrium, bone marrow-derived cells are also thought to act as endometrial stem/progenitor cells [32,33].

Among these cells, ESP cells are perhaps the most extensively studied stem/progenitor cells derived from the endometrium [34,35,36,37,38]. Treatment with Hoechst 33,342 dye followed by flow cytometric analysis enables us to identify primitive and undifferentiated cells in many species [39]. The primitive fraction has been termed the “side population” (SP) because it shows low fluorescence and consequent offset position in flow cytometric dot plots [40]. The low fluorescence results from the continuous removal of Hoechst dye via highly expressed cell membrane transporters, including adenosine triphosphate (ATP)-binding cassette subfamily G member 2 (ABCG2) [40]. Adult stem cells in many different types of tissues, organs, and species are found in the SP fraction [39].

**Figure 2:**
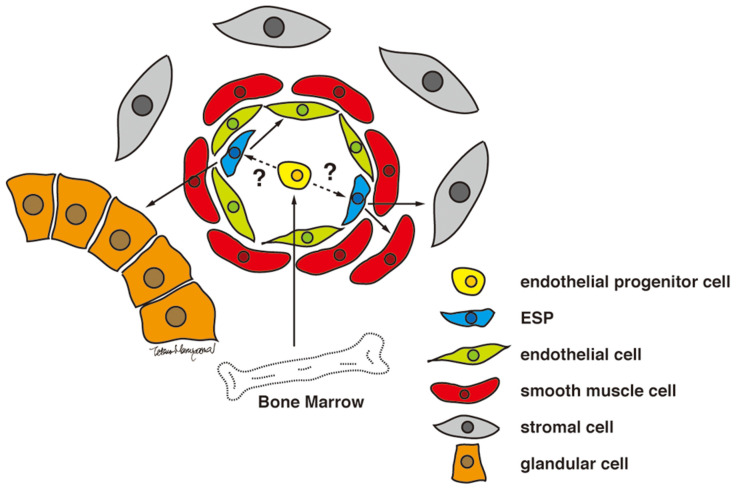
Proposed model for ESP-driven endometrial regeneration and the establishment and progression of endometriosis. Reproduced from [36] under a CC BY license, with permission from PLOS ONE, original copyright 2010.

Human endometrium contains small numbers of SP cells (approximately 3% of endometrial cell suspensions) with stem cell properties, including multi-lineage differentiation into a variety of endometrial cell components, including epithelial, stromal, endothelial, smooth muscle cells, and tissues when transplanted under the kidney capsule of immune-deficient mice [36] (Figure 2). As illustrated in Figure 1, endometrial cells positive for ABCG2, a representative marker of SP cells, are localized in the vascular wall of capillaries and small vessels in both the functionalis and basalis layers of the human endometrium [36]. Because ABCG2 is a potent cell membrane transporter capable of pumping toxicants and harmful products of oxidative stress from the inside to the outside of a cell [41], expression of ABCG2 may endow ESP cells with a robust, protective, and defensive phenotype necessary for stem/progenitor cell properties. Colocalization of ABCG2 and CD31, an endothelial marker, supports the endothelial phenotype of the ESP. ESP cells also express estrogen receptor (ER)-β, but not ER-α or progesterone receptor (PR) [36], further indicating the endothelium-like characteristics of ESP because ER-β is expressed only in endometrial endothelial cells [42].

Analysis of the surface phenotype using specific markers for endometrial cell types reveals that ESP cell are partly positive for epithelial, stromal, and endothelial markers [36]. Taken together with in vivo and in vitro multi-lineage differentiation, these findings suggest that ESP cells may include multiple stem/progenitor cell types. In particular, endothelial and endometrial mesenchymal stem cells (MSCs) (CD140b^+^CD146^+^) are enriched in the ESP fraction [38]. Nevertheless, the optimal culture system and conditions for single cell tracking and single cell differentiation assays have not been identified. Thus, one cannot exclude an alternative possibility that ESP cells are enriched for a single multipotent stem cell capable of differentiation into a variety of endometrial cell types.

**Figure 3:**
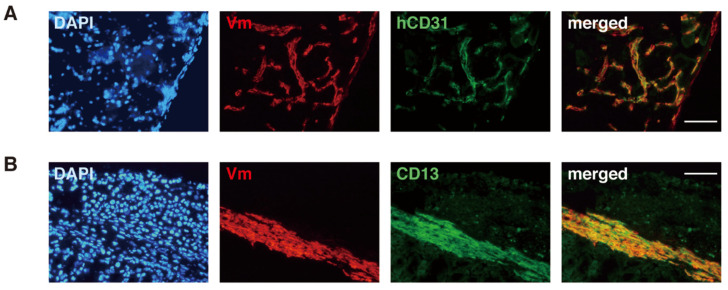
Expression of endothelial and stromal cell markers (hCD31 and CD13, respectively) in human-derived cells present around an ESP-initiated lesion. Immunofluorescence images of the ESP-initiated lesion in immunodeficient mouse kidney co-stained with DAPI and antibodies against only human vimentin (Vm) and hCD31 (**A**) or antibodies against Vm and CD13 (**B**). Bars, 100 µm. Reproduced from [36] under a CC BY license, with permission from PLOS ONE, original copyright 2010.

ESP cells are able to migrate into the mouse kidney parenchyma and form blood vessels positive for CD31 (Figure 3) when transplanted beneath the mouse kidney of immunodeficient mice [36]. The invasive and migratory potentials of ESP cells substantiate that they are a likely candidate for endometriosis-initiating cells [4], as discussed later.

## 3. The Initial Stem Cell Theory for the Pathogenesis of Endometriosis

### 3.1. Summary of the Initial Stem Cell Theory

**Figure 4:**
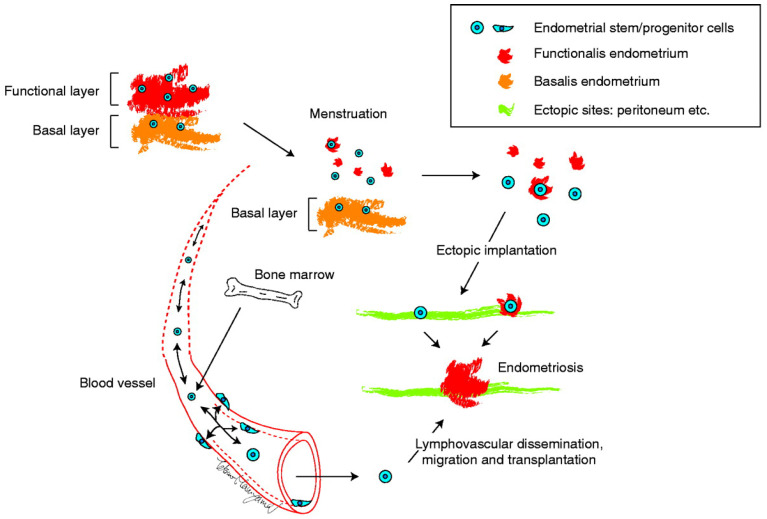
The initial proposed model for ectopic implantation of endometrial stem/progenitor cells via fallopian tubes or lymphovascular routes and subsequent establishment of the endometriotic lesion. Adapted from [16] with permission from the Society for Reproduction and Fertility.

Identification and characterization of several populations of multipotent stem cells, including epithelial-derived, mesenchymal, and a mixed side population in the endometrium and in menstrual blood led to the stem cell origin hypothesis. That is, that endometriotic lesions arise from differentiation of a single or a group of progenitor/stem cells originating from neonatal/adult endometrium, bone marrow, or Müllerian duct derivatives in a process of coelemic metaplasia [4,15,16,21,43,44]. Regardless of the site of stem cell origin, these stem cell theories collectively proposed that stem/progenitor cells translocate into/onto ectopic sites through fallopian tube-mediated retrograde menstruation, lymphovascular circulation, and/or direct migration/invasion. Subsequently, they give rise to the various types of cells observed in endometriotic lesions, i.e., glandular, stromal, endothelial, and smooth muscle cells, through differentiation, epithelial-mesenchymal transition (EMT)/mesenchymal- epithelial transition (MET), and metaplasia, as illustrated in Figure 4.

The endometrial stem cell theory is consistent with the retrograde menstruation theory, the lymphatic and vascular metastasis theory, and the iatrogenic direct implantation theory. It is likely that stem cells of endometrial origin may enter the circulatory system passively upon shedding during menstruation and find environmentally suitable sites to give rise to deep infiltrating endometriosis (DIE) and endometriosis outside the abdominal cavity including lung endometriosis. Furthermore, the stem cell theory with a wider interpretation can explain the coelomic metaplasia theory and embryonic rest theory. In other words, they posit that multipotent stem or progenitor cells with a commitment to endometrial cells aberrantly migrate and stay in various ectopic sites including mesothelium and peritoneum during fetal development.

### 3.2. Supportive Evidence for the Stem Cell Theory

There is increasing evidence to support the stem cell hypothesis. First, based on the stem cell theory, primitive endometriosis-initiating cells should have stem cell properties, including expression of several stem cell markers, and clonal expansion of these cells should generate endometriotic lesions containing some cells that (partially) express their original stem cell markers. It has been shown that cells present not only in the endometrium but also in or adjacent to endometriotic lesions express several stem cell markers, including OCT4/POU5F1, Musashi-1, and ABCG2 [45,46,47,48,49].

Second, the stem cell theory predicts that endometriotic lesions should contain a stem cell-like cell population. Chan et al. reported that ovarian endometriomas contain a subset of cells displaying a number of somatic stem cell properties. They include colony-forming activity, self-renewal capacity, and multipotency [50].

Third, stem cell origin implies clonality. For example, several earlier studies demonstrated that ovarian endometriomas are in fact monoclonal in origin [51,52,53]. These molecular findings support the single-cell derivation of endometriomas. However, the developmental history of endometriotic lesions can be more complex. For example, whereas peritoneal endometriotic lesions are polyclonal [54,55], individual glands of endometriotic lesions are monoclonal [54]. Thus, either single or multiple precursors might give rise to a single peritoneal endometriotic lesion, while the glands arise individually from a single (stem/progenitor) cell [54]. Nevertheless, a clear conclusion regarding a putative clonal process in the development of endometriosis was not included in the initial stem theory [4,15,16,21].

### 3.3. The Strength and Weakness of Stem Cell Theory

The stem cell theory improves upon the weaknesses of conventional theories and also addresses the unique characteristics and behavior of endometriosis. For instance, the retrograde menstruation theory has several shortcomings. Specifically, despite the frequent occurrence of retrograde menstruation and the high prevalence of peritoneal endometriosis, it has been extremely difficult to detect the initial pathological steps, i.e., the attachment of endometrial tissue to the peritoneum and its secondary proliferation and invasion [3,11,12]. Again, the stem cell theory posits that endometriosis arises from a single or very few endometrial stem/progenitor cells. Therefore, if a small number of stem cells (and not endometrial tissue fragments) could initiate endometriotic lesions through generation of individual endometrial cell components, detection of the initial event would be exceedingly difficult. Only after completion of the initial events would endometriotic lesions become microscopically detectable. Notably, non-stem/progenitor cells in the endometrium (the bulk of endometrial cells) may not give rise to “persistent” endometriosis even when a large number of these cells are present ectopically.

Because of the low proportion of endometrial stem/progenitor cells, for instance ESP cells (approximately 2–3% of total endometrial cells) [38], there should be very little chance that endometrial stem/progenitor cells could find a supportive microenvironment at ectopic sites, survive there, and initiate an endometriotic lesion. This might explain the apparent discrepancy between the frequencies of endometriosis and retrograde menstruation [56]. One might even speculate that endometrial stem/progenitor cells that were lost from the endometrial vascular walls during shedding might enter the circulation and migrate into ectopic sites, producing extrapelvic endometriotic lesions.

The weakness of this stem theory is that the (stem) cells responsible for the genesis of endometriotic lesions have not yet been identified. Such cells, however, should have several stem cell properties to give rise to sustainable endometriotic lesions. These properties include multipotency, vasculoangiogenesis, invasiveness, and reduced tendency for apoptosis.

### 3.4. Candidate Stem/Progenitor Cells Responsible for Genesis of Endometriosis

It is likely that several steps are required for endometriosis to evolve from a single or a few stem/progenitor cells derived from the endometrium and/or bone marrow. Endometriosis-initiating cells should have the following properties.

First, they should be present within the functional layer, i.e., tissue that is translocated during retrograde menstruation (implantation theory). Second, they should be able to attach, migrate, and initiate angiogenesis at an ectopic site. Third, the cells should possess the capability to differentiate into multiple lineages in order to reconstitute the endometrium at an ectopic site. In this context, endometriosis-initiating cells should have at least some stem/progenitor cell properties.

ESP cells as identified by my group and others [34,35,36,37,38] satisfy most of the three above-mentioned criteria of endometriosis-initiating cells. That is, ESP cells are present in the functional layer and have migratory, angiogenic, and stem cell-like properties [34,35,36,37,38], suggesting the possible potential of ESP cells to initiate endometriosis [4,16]. Thus, the presence and characteristics of ESP cells lend further credence to the stem cell theory of endometriosis and provide additional support for the retrograde menstruation theory.

ESP cells share many properties with endothelial progenitor cells. Those cells may originate from the bone marrow. Whereas ESP cells are present in the eutopic endometrium, it is likely that the origin of endometrial stem/progenitor cells may be the bone marrow as illustrated in Figure 4. ER-β is not a stem cell marker but it is expressed abundantly in endometriotic lesions [5]. Because ESP selectively express ABCG2 and ER-β [36], it is highly likely that ESP cells are a candidate for endometriosis-initiating cells.

Alternatively, if there are two or more progenitor cells that participate in the genesis of endometriosis, they should not necessarily be required to have multi-lineage differentiation potentials to generate both glandular and stromal cell components. In this regard, in addition to ESP cells, pericytes, MSC/SUSD2, and epithelial progenitor cells positive for either N-cadherin or SSEA-1 or both, as illustrated in Figure 1, are candidate(s) for endometriosis-initiating cells. This possibility will be discussed in the next section.

## 4. Recent Somatic Mutation Analyses Using Next-Generation Sequencing of Deep Infiltrating Endometriosis, Endometrioma, and Endometrium

From the mid-1990s to the mid-2000s, several groups performed analyses to identify the origin of endometrioma and peritoneal endometriosis. They used phosphoglycerate kinase (PGK) and/or human androgen receptor (HUMARA) clonality assays in combination with or without microdissection [51,52,53,54]. Those studies collectively suggested that either single or multiple precursors might give rise to a single peritoneal endometriotic lesion, whereas the glands might arise individually from single (stem/progenitor) cells [54]. The conventional clonal analyses, however, have limitations, including low resolution that limits definitive conclusions regarding the clonality and origin of endometriotic cell components.

Recent somatic mutation analysis combined with the use of microdissection and next-generation sequencing have led to new insights into the origin, clonality, and genesis of endometriosis. In 2017, Anglesio et al. [57] employed next-generation sequencing and discovered the frequent presence of cancer-associated driver mutations (CAMs) that initiate the genesis and development of tumors in the epithelial components of DIE. They also uncovered missense and synonymous passenger mutations that are unrelated to the selective growth of the cells containing these harmless mutations in DIE. Importantly, they found that the CAMs and passenger mutations were confined to the epithelial but not stromal components of DIE, suggesting a dual cell origin of DIE [57]. Otherwise, the same mutations should be present in both epithelial and stromal cell components if both cells have the same cell origin.

In general, CAMs are not the best indicator for tracing clonality because they likely influence cell survival and proliferation, i.e., clonal selection and expansion, thereby affecting cell clonality [58]. Furthermore, CAMs preferentially take place in (transformed) epithelial cells and not in stromal neoplasms [58]. In contrast, the harmless private passenger mutations are superior clonality markers because they randomly occur and are functionally silent (neutral) [58].

Taking advantage of these passenger mutations together with laser-capture microdissection, Noë et al. [59] isolated epithelial and stromal cell components separately from within the same DIE lesions. They subsequently determined the clonality of DIE lesions, focusing on mutant allele frequencies (MAFs) of individual mutations that were thought to reflect the clonal status of each component [59]. They found that these synonymous or missense passenger mutations were present only in the epithelial component and not in the stromal component from the same lesions [59]. These results further support the conclusions drawn by the previous work [57], suggesting that the epithelium is clonally and probably developmentally distinct from the stroma in the same endometriotic lesion.

More recently, Suda et al. reported that a similar repertoire of somatic CAMs was detected both in epithelial cells from ovarian endometriotic cysts and in individual endometrial glands isolated from normal uterine endometrium [60]. Based on the results, they proposed that endometriotic cysts follow an evolutionary path in which clonal expansion of epithelial cells with CAMs takes place in normal endometrial glands first, after which these cells are transported from the uterine cavity to the surface epithelium of the ovary through retrograde menstruation, resulting in the genesis of ovarian endometriotic cysts [60]. The same group subsequently analyzed an individual 56-year-old patient using multiregional whole-exome sequencing and microdissection techniques. They demonstrated that epithelial samples from uterine endometrium, ovarian endometriotic lesions distant from and adjacent to the ovarian carcinoma and the ovarian carcinoma were derived from a single ancestral clone. Those data suggest that epithelial cells of ovarian endometriosis and clear cell carcinoma were descendants of uterine endometrial epithelium [61]. Again, no mutations were shared by the stromal component of the carcinoma and epithelial components, consistent with previous studies showing different mutation profiles between the epithelium and stroma in endometriosis and uterine endometrium [61].

## 5. Modification/Revision of the Stem Cell Theory

### 5.1. Dual Cell Origin Hypothesis

The series of mutational and clonal analyses described herein collectively indicate that the evolution of both superficial and non-superficial endometriosis is complex in that the epithelium is clonal and its development is independent of stroma [59]. Based on these findings, it seems highly unlikely that endometriosis arises from a single stem or progenitor cell capable of differentiating into both epithelial and stromal cells or that epithelial cells differentiate into stromal cells through the EMT or MET [62].

Instead, to address the possibility of dual or multiple cell origins of DIE, the following evolutionary path has been proposed [62]. First, a single circulating endometrial epithelial progenitor cell translocates from either the eutopic endometrium or bone marrow to the prospective endometriotic site and then clonally expands transiently to generate a budding endometrial gland. Subsequently, circulating mesenchymal stem cells or endometrial stromal progenitor cells are recruited to the endometriotic lesion that initially contains only glandular cells. The result is the formation of an endometriotic lesion consisting of both epithelial and stromal cells [62].

In this scenario, CAMs can occur in epithelial cell components at any time, i.e., before, during, or after the establishment of endometriotic lesions. However, considering the data provided by Suda et al. [60,63], it is more likely that epithelial CAMs are already present in the eutopic endometrium prior to the establishment of endometriotic lesions, after which epithelial CAMs are further accumulated through clonal selection and expansion during the establishment of endometriotic lesions. Unlike the epithelial component, the accumulation and persistent presence of CAMs are extremely rare in the stromal component because the stromal cells are polyclonal, i.e., are derived from different progenitor cells [62].

The recruitment of endometrial cells, including endometrial stem/progenitor cells, into preexisting endometriotic lesions is an entirely reasonable hypothesis because endometriotic lesions themselves produce chemokines and cytokines that attract eutopic endometrial cells as well as immune cells, the latter of which, in turn, further secrete a variety of cytokines and chemokines [64]. In support of this concept, in a mouse model of retrograde menstruation, uterine cells freshly introduced into the peritoneal cavity can successfully integrate and contribute to various cell populations within preexisting endometriotic lesions [65].

### 5.2. Possible Evolutionary Paths Based on the Dual Cell Origin Theory

**Figure 5:**
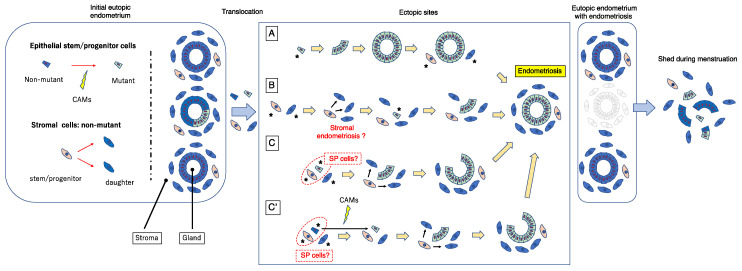
A revised stem cell theory that proposes a cellular model for the pathogenesis of endometriosis. Blue and yellow green quadrangles represent endometrial epithelial stem/progenitor cells without or with CAMs, respectively. Light orange and blue snap pea-like shapes represent endometrial stromal stem/progenitor cells and their daughter cells. Red arrows indicate the progressive generation from the left to right. The asterisks indicate endometrial cells that are derived from eutopic endometrium and implanted ectopically via retrograde menstruation and/or vascular lymphatic dissemination. The detailed explanation of this figure is provided in the text. SP, side population; CAMs, cancer-associated mutations.

The above scenario for the genesis of endometriosis is attractive and entirely possible, but, based on the dual cell origin hypothesis, I herein propose a possible alternative mechanism as illustrated in Figure 5. CAMs can develop in glandular and/or luminal epithelial (stem/progenitor) cells of the eutopic endometrium, as suggested by the previous studies [60,63,66]. One or a few endometrial glandular and/or stromal stem/progenitor cells together with or without mature endometrial cells/tissue could translocate into ectopic sites via retrograde menstruation and/or vascular lymphatic dissemination. Several mechanisms are possible (Figure 5). To simplify the pathologic mechanism, mature endometrial glandular cells are minimally illustrated in Figure 5.

In pattern (A), a single or a few CAM-bearing endometrial glandular epithelial cells translocate onto an ectopic site. Subsequently, they generate mutant endometrial gland(s) through clonal expansion, and thereafter recruit endometrial stromal stem/progenitor cells to surround and support the gland. This scenario has been proposed by Shih et al. [62] and is described in the previous section. Although this mechanism is attractive and entirely possible, one can argue that endometriotic lesions are usually accompanied by stromal components. Indeed, occult endometriosis, which is thought to be a lesion at a very early stage of endometriosis, is always accompanied by stromal components [67]. Furthermore, human endometrial glandular cells alone prove difficult to maintain in long-term culture, and the in vivo features of these cells have not been sufficiently recapitulated in culture systems, including three-dimensional (3D) cultures [68,69]. Recently, hormone-responsive epithelial organoids have been successfully established with the use of 3D uterine epithelial cultures [70,71,72,73,74]. However, the culture media and protocol underlying these 3D culture systems are ideally designed to reproduce the eutopic endometrial microenvironment. It is likely that ectopic sites, including the peritoneum and the surface of the ovary, may not be suitable for one or a few epithelial stem/progenitor cells even with CAMs to survive, grow, and form well-structured glands. Indeed, when endometrial SP cells containing endometrial epithelial stem/progenitor cells are transplanted under the kidney capsule of immunodeficient mice, they show little capacity to generate endometrium-like tissues with a glandular structure [36]. However, they are able to generate the entire endometrium efficiently in the presence of supporting cells [38].

In pattern (B), a single or a few non-mutated endometrial stromal stem/progenitor cells alone initially translocate onto ectopic site(s) and generate the stromal component of endometriotic lesions. These stromal lesions subsequently recruit a single or few endometrial glandular epithelial stem/progenitor cells harboring CAMs that in turn colonize and expand, thereby giving rise to mutated endometrial glands. In this way, endometriotic lesions consisting of non-mutated stromal cells and mutated glandular cells could be established. If ectopic endometrial stromal cells fail to recruit endometrial glandular epithelial stem/progenitor cells, the stromal cells alone may colonize and expand without endometrial glands and finally give rise to “stromal endometriosis” in which endometriotic lesions contain stromal cell components but lack glandular components [75,76]. Indeed, stromal endometriosis is rather often observed [75,76], whereas, to my knowledge, endometriosis without stromal cell components but only with glandular cell components is extremely rare. In support of this pattern, occult endometriosis, which is thought to be a lesion at the very early stage of endometriosis, is always accompanied by stromal components [67].

In pattern (C), a single or a small number of endometrial glandular epithelial stem/progenitor cells harboring CAMs and non-mutated endometrial stromal stem/progenitor cells simultaneously translocate to ectopic sites. The former epithelial cells exhibit clonal expansion resulting in the generation of a CAM-bearing endometrial gland and the latter give rise to non-mutated stromal cells to surround and support the mutant endometrial gland. Because endometrial SP cells are able to generate endometrial glands and stroma [34,35,36,37,38], it is possible that SP cells could be endometriosis-initiating cells.

Pattern (C’) is similar to pattern (C) except that the epithelial endometriosis-initiating cells do not initially have CAMs. Subsequently, CAMs take place in the epithelium of the tentatively established endometriotic lesions as often observed in the epithelium of eutopic endometrium [60,63,66]. The CAMs-bearing epithelial stem/progenitor cells then clonally expand and generate endometriotic lesions together with non-mutated stromal stem/progenitor cells in a way similar to pattern (A), whereas non-CAMs-bearing epithelial cells do not survive and disappear eventually at the ectopic site.

In all of these pathways, the final product is an endometriotic lesion that consists of mutated glands and non-mutated stroma, consistent with previously reported data [57,59,60,61,62,63]. Importantly, when such an endometriotic lesion is established, the mutated eutopic endometrial glands responsible for the ectopic mutated glands may have already been shed and lost following a single or repeated menstruations, as illustrated in Figure 5. This could account for the low incidence of mutated eutopic glands despite the high prevalence of mutated ectopic glands, in particular DIE [57].

Importantly, the premise of patterns (B) and (C’) is the presence of pre-existing initial and tentative endometriotic lesions without CAMs before the final establishment of endometriotic lesions. Based on the theories described here, these pre-existing endometriotic lesions may not be able to maintain themselves for a prolonged period because they do not contain stem/progenitor cells. Once the initial endometriotic lesions recruit (as pattern [B]) or create (as pattern [C’]) CAMs-bearing or epigenetically changed epithelial stem/progenitor cells, they can survive and behave as active lesions similar to the red lesions of peritoneal endometriosis. Otherwise, such tentative endometriotic lesions eventually disappear, resulting in scar formation similar to the white lesion of peritoneal endometriosis.

### 5.3. Robustness of the Dual Stem Cell Theory for the Pathogenesis of Endometriosis

The presence of both driver and passenger mutations in epithelial and stromal cells is rare [57,59,60,61,62,63,66]. Consequently, single or low numbers of endometrial stem/progenitor cells with these mutations do not give rise to both epithelial and stromal cells in endometriotic lesions and endometrium. However, it is possible that a stem/progenitor cell population such as ESP cells might be capable of generating endometrial epithelial, stromal, endothelial, and smooth muscle cells [34,35,36,37,38]. In addition, putative endometrial stem cells without mutations could translocate into ectopic sites and give rise to both epithelial, stromal, and other types of endometriotic lesion cells, as illustrated in Figure 5 (pattern C’). Subsequently, driver and passenger mutations could occur in the epithelium of the newly established endometriotic lesions (pattern C’) in a similar way to the epithelium of the eutopic endometrium [60,63,66]. Because the microenvironments of ectopic sites are generally unsuitable, only epithelial stem/progenitor cells with driver mutations, i.e., CAMs, could survive, i.e., undergo clonal selection and thereafter expand in assistance with supporting stromal cells harboring no or few mutations. In contrast, epithelial stem/progenitor cells and/or glands without CAMs could not survive.

This latter scenario, however, does not apply to a rare case in which three distinct and independent DIE sites have the same KRAS mutation [57]. In this case endometrial epithelial stem/progenitor cells with CAMs present in the eutopic endometrium may be transported into three different pre-existing endometriotic lesions as either pattern of A, B, and C in Figure 5. Intriguingly, the same KRAS mutation was not detected in the eutopic endometrium in this case [57]. It is highly likely that endometrial epithelial stem/progenitor cells harboring the same KRAS mutation would disappear. This is because menstruation provokes endometrial tissue shedding, in particular the functional layer of the endometrium, resulting in the clearance of the mutated epithelial stem/progenitor cells responsible for initiating the three different endometriotic sites. Indeed, no endometrial KRAS mutation was detected in premenopausal patients [77], suggesting that menstruation probably plays an important role in the removal of KRAS mutations of the endometrium.

The dual stem cell theory seems robust, but greater elucidation of the mechanisms underlying the dual stem cell theory is needed. Furthermore, the dual stem cell theory does not exclude the possibility that endometriotic epithelial and stromal cells arise from a common hypothetical stem/progenitor cell as proposed by the initial stem cell theory [4,16]. It remains possible that these common precursor cells could give rise to two or more different types of cells with distinct patterns of genome evolution if they expanded independently of each other from a very early stage of divergence or differentiation, as suggested elsewhere [63].

## 6. Conclusions

Endometrial stem cells and their supportive niches were mostly hypothetical prior to 2000, but thereafter, through identification and characterization of those cells and systems, they have been recognized as critically important. In accordance with advances in endometrial stem cell physiology, the stem cell theory (hypothesis) for the pathogenesis of endometriosis has emerged over the past decade. The initial theory proposed that endometriosis arose from a single or a few endometrial stem/progenitor cells present in eutopic endometrium or bone marrow as a result of retrograde menstruation and/or lymphovascular dissemination. The stem cell theory may permit unification of the various theories accounting for implantation and coelomic metaplasia. More recent evidence based upon somatic mutation analyses of microdissected endometriotic and endometrial samples using next-generation sequencing has substantiated the initial stem cell theory but with some revision. At present, the most likely mechanism is that epithelial stem/progenitor cells independently acquire CAMs and that both the epithelial stem/progenitor cells with CAMs and stromal stem/progenitor cells without CAMs differentially, sequentially, but coordinately participate in the genesis of endometriotic lesions. Given that the origin of endometriosis is eutopic endometrium, the candidate cells for endometriotic epithelium-initiating cells are likely to be endometrial epithelial cells positive for either N-cadherin or SSEA-1 or both. The candidate cells for endometriotic stroma-initiating cells may be endometrial mesenchymal stem cells positive for SUSD2. ESP cells are also a possible candidate because they contain unipotent or multipotent cells capable of behaving as endometrial epithelial and stromal stem/progenitor cells. The dual stem cell origin model is particularly relevant for DIE and ovarian endometrioma, presumably via retrograde menstruation or lymphovascular dissemination. However, it remains unclear how both types of stem/progenitor cells coordinately determine their fates. Elucidation of the precise origin of endometriotic cells and possible stem cell-mediated mechanisms underlying the pathogenesis of endometriosis will contribute to the development of new modalities of diagnosis, treatment, and management of endometriosis, but such developments await further studies.

## Data Availability

Not applicable.
